# Parental Concerns Regarding Bracing Compliance for Children With Clubfoot: Seeking Support on Facebook

**DOI:** 10.7759/cureus.43761

**Published:** 2023-08-19

**Authors:** Natalie Tonkovich, Danika Baskar, Steven Frick

**Affiliations:** 1 Orthopaedic Surgery, Stanford University School of Medicine, Palo Alto, USA

**Keywords:** community, information sharing, support, social media, ponseti, talipes equinovarus, clubfoot

## Abstract

Background

Clubfoot treatment requires a period of bracing until early childhood to maintain the initial correction achieved by Ponseti casting and serial manipulations. During this period, bracing compliance is the most important factor in preventing the relapse of clubfoot deformity. This period can be challenging for parents, given several factors that affect treatment adherence. In recent years, social media has opened up new ways to seek guidance from an online community, including health-focused areas such as congenital clubfoot. This study examines bracing-related concerns that arise during clubfoot treatment that lead caregivers to seek support from online forums like Facebook.

Methodology

Six Facebook clubfoot support groups with the highest number of clubfoot posts were evaluated to identify the relative proportion and content of posts related to bracing compliance during December 2021. Bracing-related concerns across all identified posts were then organized into the following six domains that may affect the child’s bracing adherence: physical, psychological, commercial, social, bracing device-related, and parental.

Results

In December 2021, there were 442 total posts across the six clubfoot-focused Facebook groups analyzed. Of these, 23.1% of posts were directly related to bracing compliance. Approximately 22% of these posts had responses where at least one fellow parent suggested seeking advice from a healthcare professional. When these root concerns were organized into six domains that can affect the child’s bracing compliance, we found 49 physical, 26 psychological, 5 commercial, 0 social, 14 bracing device-related, and 8 parental factors.

Conclusions

In this study, 23.1% of all analyzed Facebook posts involved discussion about brace-related concerns, making this a significant topic of discussion on online parental forums. Facebook groups create a community and provide emotional support to parents that support bracing compliance. Clubfoot physicians should be aware of key parental concerns related to bracing compliance, and physicians can provide education on bracing that provides accurate information and anticipatory counseling during regular check-ups with patients and their families.

## Introduction

Ignacio Ponseti, M.D.’s method for the treatment of congenital clubfoot has become the global standard for initial correction of congenital clubfoot deformity [1]. The Ponseti method involves serial manipulations and plaster casts (usually with tendo-Achilles tenotomy) to achieve initial correction of the deformity, followed by bracing to maintain proper foot positioning [2]. The initial casting phase typically lasts seven to nine weeks and can be considered the “doctor part” of the Ponseti method treatment, while the bracing phase is much longer, typically lasting two to four years, and is considered the “parent part.” Ponseti’s method has shown the highest success rates in maintaining correction in short and long-term studies [3]. An integral part of the Ponseti treatment is adherence to brace-wearing full-time for 23 hours for three months after cast correction and thereafter at nap and nighttime until the child is up to four to five years old [2]. However, data from patients show that bracing compliance becomes more difficult as the child gets older [4]. Studies also found that non-compliance is the leading risk factor for relapse/recurrence of clubfoot deformity [4-9]. Parents have reported various challenges with bracing compliance, including finding appropriate sizing of the brace, blisters from the brace, and children not being able to sleep with the brace [10]. These issues make this parent-driven phase of treatment challenging.

During the bracing stage of treatment, parents may seek information and support from various sources, including social media platforms, as they provide readily accessible information during a stage of treatment where parents may not be taking their child in for regular visits like they were initially during the weekly casting phase [10-12]. In our previous study [10], we found a common topic of posts on Facebook was questions about bracing. Parents on these online discussion groups shared information and advice related to the bracing stage of the treatment, ranging from recommended clothing to wear with the brace, to advice on how to treat blisters resulting from brace wear. Although these online clubfoot communities have provided support to families undergoing treatment, few physicians were noted to participate in these social media sites, leading to concerns about the validity of the information being shared. This study examines the various bracing-related concerns that lead caregivers to seek guidance from an online community. We chose Facebook as the social media platform as our previous study revealed that Facebook was an interactive site for parents to seek advice from fellow parents. In contrast, Instagram and TikTok featured photos and successful treatment stories, while Twitter offered live webinars, educational resources, and professional advice.

## Materials and methods

A search for clubfoot-focused Facebook groups with the most posts per month was conducted using the search terms “clubfoot,” “talipes,” and “equinovarus” from September 1st, 2020, to October 19, 2020, and again during the month of December 2021. During the first search, five groups were identified [10]. During the second search, we confirmed that the activity in these five groups was similar to the frequency of activity during the first search. An additional group with high posts per month was identified. The six groups were then evaluated to identify all posts related to bracing compliance during December 2021.

After the posts related to bracing compliance were selected, the root concerns across all identified bracing compliance questions were organized into the following six domains that can affect the child’s bracing adherence: physical, psychological, commercial, social, bracing device-related, and parental. Responses to the original posts were also examined, including whether fellow parents recommended seeking advice from healthcare professionals. Descriptive statistics are reported.

## Results

The six groups with the highest engagement over the study period that were subsequently analyzed included Clubfoot Moms, Clubfoot and Talipes UK, Clubfoot Connection, Happy Feet Talipes New, Parent Support Group for Children/Babies With Clubfoot, and Clubfoot Baby Support Group. Out of a total of 442 posts across the six Facebook clubfoot groups, 102 posts were related to bracing compliance.

**Figure 1:**
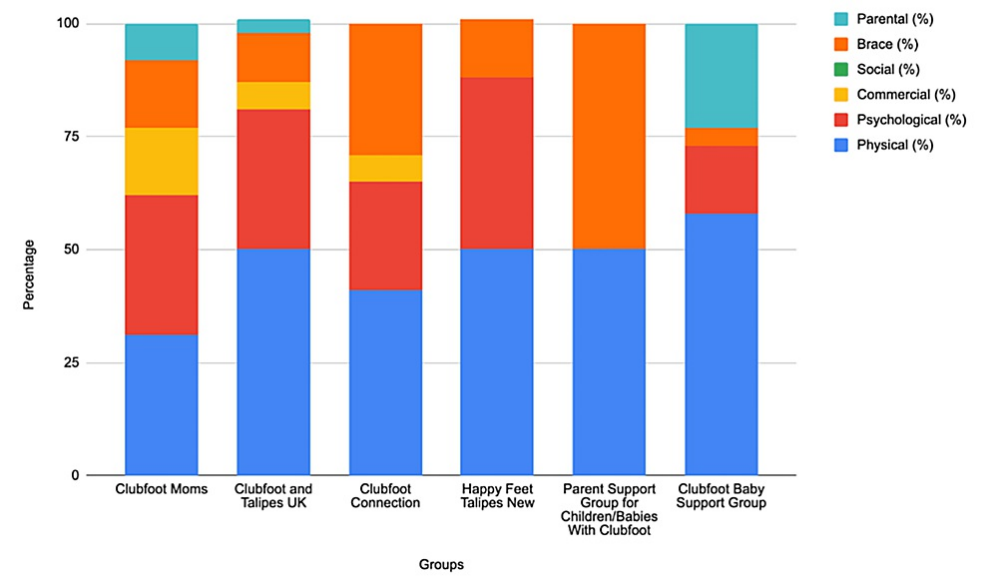
Percentage of posts that were bracing-related for each group.

The 102 posts were then organized into six domains for each group (Figure 1). All groups had posts related to physical concerns and brace-related questions. All but one group had posts related to psychological concerns.

**Figure 2:**
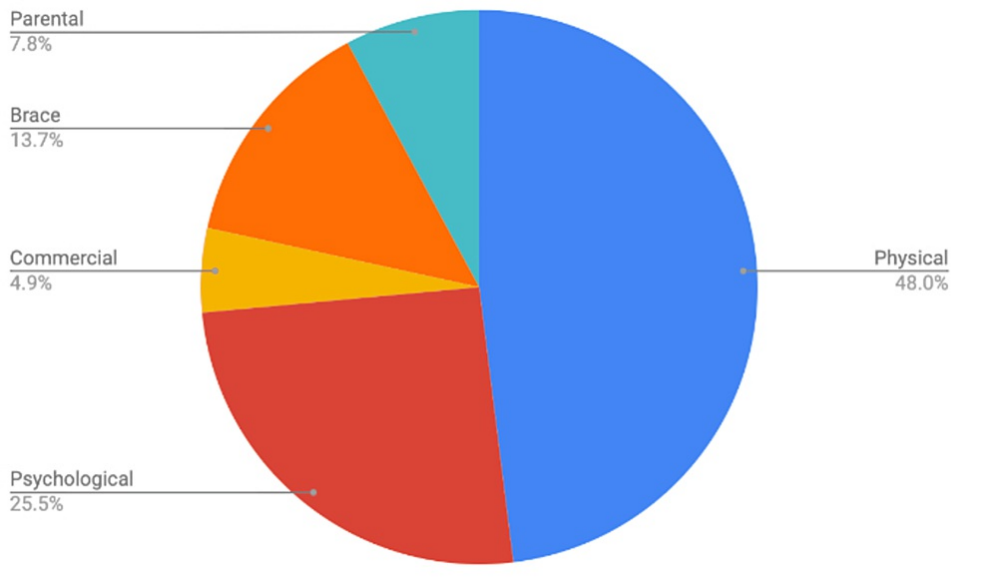
Factors affecting the child’s bracing compliance for six groups.

The total percentages for the six groups are shown in Figure 2. A total of 48% of all posts from the six groups concerned physical questions, followed by psychological (25.5%) and brace (13.7%). There were no postings related to social factors, such as the child comparing themselves to their peers or sibling.

Table 1 shows examples of each of the parental concerns across the six domains. In this study, 48% of the posts concerned physical factors, including questions about blisters or skin concerns. Parents sought advice on treating blisters or their baby’s skin discoloration. Also in this category were parents asking about nighttime kicking with the brace and what they could do about the noise from the bar hitting the crib. The second most common domain, comprising 25.5% of the total posts, was psychological, such as sleep concerns or their child resisting the brace. Parents noted that their babies seemed frustrated and upset when their braces were on or that their babies had been crying all day since they started bracing treatment. The third common topic was the bracing device itself. Parents wanted to know when the brace became too small or when and how much to lengthen the bar width between the two boots. Parental-focused factors contributed to 8% of bracing-related posts. Parents questioned if they could stop bracing earlier than advised by their physician or alter the recommended course of treatment, particularly concerning bracing hours. Lastly, there were a few posts related to commercial factors, such as which brace model they should choose or whether they should switch to a different brace model.

**Table 1:** Examples of parental concerns across the six domains.

Physical	Psychological	Brace	Parental	Commercial	Social
What should we do with blisters (or other skin conditions) on the foot? What can we do about kicking with the brace at nighttime? I’m struggling to get my child’s heel down in the brace and s/he kicked it off! My child’s toes are curling. Is this ok?	What can we do if my child cannot sleep with the brace? What can we do if my child resists the brace? What can we do when my child is frustrated and upset with the brace?	When do we know when the brace is too small? When should we lengthen the bar? What length should the bar be?	Can we stop the bracing treatment earlier than advised by the physician? Can we alter the recommended course of treatment, such as reducing the bracing hours? What are the chances that we’ll be given the OK for longer free time? We forgot to pack the bar and brace. What should we do?	Which brace model should we get? Has anyone been successful with ADM?	My child asks why his/her sister/brother/friend doesn’t have to wear a brace

Among the responses to the posts, the advice varied from comments providing emotional and validating other families’ experiences to suggestions for what had worked for their child. Some parents mentioned that their child will get used to brace-wearing in acknowledgment of how time can be an important factor in their child getting accustomed to this phase of treatment. In one of the groups, a fellow parent even included links to medical journal articles in an attempt to provide evidence-based advice. Overall, 22 posts had responses where at least one fellow parent suggested seeking advice from a healthcare professional and/or getting a second opinion. No verification of the validity of advice by a healthcare provider or someone experienced in caring for patients with clubfoot was noted across the Facebook posts and responses analyzed. There was one post in which a caregiver tagged a physician who then offered an invitation to make an appointment for a second opinion.

## Discussion

Social media is a powerful tool for sharing information and offering emotional support [13]. Through Facebook support groups, parents can ask questions and receive instantaneous feedback about various topics, including those related to a person’s health, without waiting for doctor appointments [14]. Especially considering parents of children with a condition such as congenital clubfoot, social media provides a forum for families to connect and share advice with one another [10]. Parents can create a much-needed community and support system through their discussions and interactions. This support system may be helpful to parents as they navigate the recommended four to five years of bracing, where the intervention adherence becomes largely driven by parents and families caring for a child with clubfoot.

Many questions shared by parents on the Facebook groups analyzed in this study seek information on physical concerns, accounting for 48% of the total posts related to bracing. The next most common set of questions asked by parent members across the groups pertained to psychological concerns, with questions focused on their child resisting the brace, seeming frustrated, upset, or crying when the brace was on, and their difficulty sleeping. Bracing device and commercially related factors were additionally a subset of questions posted in the groups, including inquiries about recommended bar length and when to size up to the next boot size. Parents were also noted to share information from what they had been personally advised by their own child’s physician, in addition to guidance from experiences caring for their child. These types of questions could be addressed in a brochure and reinforced by physicians during regular check-ups to ensure that parents get accurate and up-to-date information. Moreover, 33.3% of posts concerned psychological and parental factors that may require answers from healthcare professionals and may not be appropriately answered by fellow parents who do not have medical training. Common concerns, such as a child resisting the brace, may have underlying medical issues, or a child may be going through a relapse that would require medical treatment that only a physician can provide. Questions about ending brace-wear earlier than recommended or what to do when parents forget to bring the child’s boots and brace during vacation may also be best answered by their child’s physician, who has examined the child’s feet.

As has been shown above, given that the bracing phase of treatment is a parent-driven intervention phase of the treatment [15], it is a significant time during which parents reach out to others on social media for support. The advice provided on these sites is mostly from personal experience and should be taken with caution, given it does not come from a child’s healthcare provider. In fact, only 22 out of the 102 posts had a fellow parent suggest seeking advice from a healthcare professional or getting a second opinion. Strategies to improve communication between clubfoot experts and caregivers are needed, with emphasis by healthcare teams on providing families with reliable sources of information, including discussion about how to analyze and interpret information from internet and social media sources. Parents may proactively ask their child’s healthcare provider regarding possible challenges with the bracing phase of treatment and should be encouraged to reach out to their child’s healthcare provider when brace compliance becomes difficult. Additionally, healthcare providers caring for children with clubfoot may address commonly asked questions in informational brochures that can be reinforced and revisited during appointments to ensure that parents get accurate and up-to-date information. Helping families establish communication via existing secure clinical messaging systems allows parents to send significant questions regarding their child to the care team. This not only creates another line of support for families but also allows clinicians to triage questions that may require further evaluation and can help facilitate scheduling an appointment.

It should be noted that this study does have limitations. Given that we analyzed posts from six Facebook clubfoot-support groups, it is possible that other categories of bracing-related questions or concerns from parents may be discussed in other groups which were not captured in our data. Also, as the study analysis period occurred during a time when several precautions were still in place as a result of the COVID-19 pandemic, it is possible that the types of questions and concerns being shared during the study period may be different when compared to other time points, especially if visits with healthcare providers were approached with caution based on need or in an effort to avoid exposure. A possible area of future study may include observing whether parents visited a physician for their concerns about their child’s clubfoot brace after initially inquiring on Facebook.

## Conclusions

Social media groups allow parents of children with congenital clubfoot to support each other and foster community-building. While much of the advice shared on Facebook clubfoot support groups comes from the experience of caring for an affected child, this information has great value in providing emotional support and practical guidance coming from caregivers who have undergone similar experiences and can relate to challenges currently faced by parents. In our study, we found that only 22% of the Facebook posts suggested that parents consult a healthcare professional. Clubfoot physicians should be aware that social media can be a potential source for parents seeking information about this condition and may want to caution families about the nature of this advice. Clinicians can address key parental concerns related to bracing compliance by engaging in proactive discussion to better support clubfoot families while transitioning to and maintaining successful adherence to their child’s bracing phase of clubfoot treatment.
